# Stimuli-Responsive Cationic Lyotropic Liquid Crystalline Nanoparticles: Formulation Process, Physicochemical and Morphological Evaluation

**DOI:** 10.3390/pharmaceutics17091199

**Published:** 2025-09-15

**Authors:** Maria Chountoulesi, Natassa Pippa, Varvara Chrysostomou, Aleksander Forys, Barbara Trzebicka, Stergios Pispas, Costas Demetzos

**Affiliations:** 1 Section of Pharmaceutical Technology, Department of Pharmacy, School of Health Sciences, National and Kapodistrian University of Athens, Panepistimioupolis Zografou, 15771 Athens, Greece; natpippa@pharm.uoa.gr (N.P.); vchrysost@pharm.uoa.gr (V.C.); demetzos@pharm.uoa.gr (C.D.); 2 Theoretical and Physical Chemistry Institute, National Hellenic Research Foundation, 48 Vassileos Constantinou Avenue, 11635 Athens, Greece; pispas@eie.gr; 3 Centre of Polymer and Carbon Materials, Polish Academy of Sciences, 34 ul. M. Curie-Skłodowskiej, 41-819 Zabrze, Poland; aforys@cmpw-pan.edu.pl (A.F.); btrzebicka@cmpw-pan.edu.pl (B.T.)

**Keywords:** lyotropic liquid crystals, drug delivery nanosystems, cubosomes, cryo-TEM, resveratrol, phytantriol, tri-phenyl-phosphine cation (TPP), DMAEMA, pH-responsive

## Abstract

**Background/Objectives**: Lyotropic liquid crystalline nanoparticles are promising drug delivery nanocarriers, exhibiting significant technological advantages, such as their extended internal morphology. In this study, cationic non-lamellar lyotropic–lipidic liquid crystalline nanoparticles were formulated by phytantriol lipid. **Methods**: The poly(2-(dimethylamino)ethyl methacrylate)-b-poly(lauryl methacrylate) block copolymer carrying tri-phenyl-phosphine cations (TPP-QPDMAEMA-b-PLMA), was employed as a stabilizer co-assisted by other polymeric guests. The exact qualitative and quantitative formulation of the systems was investigated. Their physicochemical profile was depicted from a variety of light scattering techniques, while their microenvironmental parameters were determined by fluorescence spectroscopy using adequate probe molecules. The effect of environmental conditions was monitored, confirming stimuli-responsiveness properties. Their morphology was illustrated by cryo-TEM, revealing expanded internal assemblies. Resveratrol was incorporated into the nanoparticles and the entrapment efficiency was calculated. **Results**: Their properties were found to be dependent on the formulation characteristics, such as the lipid used, as well as the architecture of the polymeric stabilizer, also being found to be stealth toward proteins, exhibiting stimuli responsiveness and high entrapment efficiency. **Conclusions**: The studied liquid crystalline nanoparticles, being stimuli-responsive, with high cationic potential, high loading capacity and showing intriguing 3D structures, are suitable for pharmaceutical applications.

## 1. Introduction

Lyotropic liquid crystalline nanoparticles can be utilized as drug delivery nanocarriers exhibiting significant technological advantages, such as their extended internal morphology of a 3D- or 2D-structured network of lipid bilayer channels embedding water that are ordered either in cubic (forming the cubosomes) or hexagonal (forming the hexosomes) mesophases [1,2,3,4]. The aforementioned morphological characteristics allow advanced properties in terms of increased high drug entrapment efficiency (more than that of micelles or empty lipid vesicles, like liposomes), the ability of modifying drug release kinetics and functional versatility, and greater membrane stability than liposomes because of their compact conformation [5,6,7,8,9,10,11,12]. Further, the reduced toxicity, biodegradability and biocompatibility of the lipids utilized, like phytantriol and glycerol monooleate, reinforces their applicability to the nanomedicine field [13,14].

The stability of the non-lamellar internal structure is ensured by polymeric stabilizers that intercalate within the lipid bilayer. The mode of the lipid–polymer interactions strictly affects the physicochemical and the morphological characteristics, like the internal architecture, the drug entrapment efficiency, the exposure mode to biological barriers or cells or proteins, and thus the final pharmacokinetic and drug release profiles and bioavailability [15,16]. Despite the efforts having been already reported in the current prior art [17,18,19,20,21,22,23], there is a continuously emerging need for nanoformulations of liquid crystals with a variety of properties, including bio-adhesiveness, tissue penetration, formulation simplicity, flexible loading capacity and stability at various dilution environments, in order to be administered by different routes, including transdermal, intravenous, oral, or even topical pathways. Toward this scope, different lipid combinations should be further investigated, by also exploiting the field of polymer chemistry toward new and multi-functional polymeric stabilizers.

Phytantriol (PHYT) is a commercially available lipid, known to be used industrially in cosmetics, like skincare products, and identified to form non-lamellar lyotropic phases according to its phase diagram. One of the typical advantages of phytantriol over other fatty acid-based materials like glyceryl monooleate, is its improved chemical stability in aqueous and model gastrointestinal conditions. This advantage results from the absence of ester and unsaturated bonds, providing resistance to degradation phenomena [24,25]. Phytantriol has also been found to exhibit higher resistance to the gastric environment, making it more suitable for drug oral administration [26].

Although the combination of phytantriol with quaternized copolymers is not widespread, thus highlighting the novelty of the present study, there some studies reported in the literature that employed other additives—cationic amphiphiles, such as didodecyldimethylammonium bromide (DDAB) [27] or cetrimonium bromide [28], which can affect the phase behavior and stability of PHYT-based cubosomes. However, in these studies, the addition of the amphiphilic yielded various phase cubic–cubic transitions, which gradually affected the stability of the inner structure. Therefore, a cationic stabilizer that does not alter the inner symmetry is still needed. Conversely, there are some recent examples of pH-sensitive formulations, referring to glyceryl monooleate lipid, like the SN-38-loaded pH-sensitive lipid nanoparticles from glyceryl monooleate [29] or the mixed monoolein (GMO)/2-hydroxyoleic acid [30], that were found to be possible candidates for anticancer delivery to tumor sites, and pH-sensitive mucoadhesive cubosomal monoolein lipid/polyelectrolyte nanocomplexes for oral administration [31]. The present study aims to fill the literature gap regarding the development of novel pH-sensitive nanosystems, exploiting the advantages of both the PHYT lipid and polymer chemistry.

The scope of the present study is the development of new lyotropic lipidic liquid crystalline nanoparticles, prepared from phytantriol lipid, with stimuli-responsiveness (pH- and thermo-), stealthiness and highly organized internal morphologies for enhanced loading capacity. Toward this scope, we incorporated the amphiphilic block copolymer poly(2-(dimethylamino)ethyl methacrylate)-b-poly(lauryl methacrylate) (TPP-QPDMAEMA-b-PLMA) as polymeric stabilizer, bring partially quaternized by the delocalized cation TPP. The stabilizing ability of TPP-QPDMAEMA-b-PLMA was evaluated in two percentages, while TPP-QPDMAEMA-b-PLMA was also co-assisted by other stabilizers, namely Poloxamer P407, two poly(ethylene oxide)-block-poly(ε-caprolactone) (PEO-b-PCL) copolymers, and two poly(2-methyl-2-oxazoline)-grad-poly(2-phenyl-2-oxazoline) (MPOx) gradient copolymers, which all exhibit high biocompatibility, low toxicity and promising stealth properties.

In more detail, colloidal dispersions of liquid crystalline nanoparticles from phytantriol lipid were developed, following an effective formulation process, in order to assess the exact qualitative and quantitative characteristics of the formulation, like the copolymer combination and the lipid-to-polymer ratio. Light scattering techniques (dynamic, static and electrophoretic) were applied in physicochemical and morphological terms. Additionally, the effects of pH, temperature, serum proteins and ionic strength were monitored. Cryo-TEM was applied for the morphological illustration of the systems’ internal structure. Moreover, their microenvironmental parameters, micropolarity and microfluidity, were investigated by fluorescence spectroscopy. Finally, the entrapment of the hydrophobic drug molecule resveratrol was attempted, and the entrapment efficiency (EE%) was assessed.

To the best of our knowledge, this is the first report on liquid crystalline nanoparticles comprising phytantriol lipid and quaternized copolymer, intended to provide stimuli-responsive cationic carriers and to elucidate the phytantriol–copolymer interactions, as a contribution to the improvement in liquid crystalline drug delivery systems. This newly proposed PHYT lipid–copolymer combination yielded to first-appeared morphological patterns, being ideal for drug loading, along with some extra provoked morphological characteristics that reinforce the advantages of the liquid crystalline nanoparticles described above, such as the loading capacity. These differences can be attributed to the differentiated lipid, i.e., the PHYT. Thus, this newly proposed PHYT lipid–copolymer combination provides novel formulations for drug delivery with upgraded functionalities.

## 2. Materials and Methods

### 2.1. Materials

Phytantriol was purchased from DSM Nutritional products Ltd. (Heerlen, The Netherlands). The employed copolymers were synthesized as described below, while Pluronic^®^ F-127 (Poloxamer P407) (PEO_98_-PPO_67_-PEO_98_), with an average molecular weight of 12,600 g/mol, and resveratrol, were acquired from Sigma-Aldrich Chemical Co (St. Louis, MO, USA). HPLC-grade water was used during preparations, while all the other materials used were obtained from Sigma-Aldrich Chemical Co.

### 2.2. Methods

#### 2.2.1. Synthesis of Copolymers

The synthesis of the copolymers is analytically described by [32,33,34] and their characteristics are depicted in Table 1 and Figure S1. The synthesis of PEO-b-PCL copolymers is described in [33] and that of MPOx copolymers in [34].

In the case of the TPP-PDMAEMA-b-PLMA, the reversible addition fragmentation chain transfer polymerization (RAFT) process was applied for PDMAEMA-b-PLMA synthesis, with a two-step polymerization procedure [35]. The tertiary amine group of the PDMAEMA was partially quaternized with 4-bromobutyl triphenyl phosphonium bromide (more details are provided in Supplementary Materials). The quaternization degree was 20%, as measured by ^1^H NMR spectroscopy.

#### 2.2.2. Preparation Process of the Liquid Crystalline Nanoparticle Dispersion

First, two different weight ratios with TPP-QPDMAEMA-b-PLMA were prepared, lipid:polymer 9:1 and 4:1, which are 10% and 20% *w*/*w* copolymer percentage, respectively.

Subsequently, the TPP-QPDMAEMA-b-PLMA stabilizer was combined with P407, PEO-b-PCL H1, H4, MPOx1 and MPOx2, in 20% *w*/*w* total copolymers percentage. The lipid concentration was 20 mg/mL in all prepared systems. The formulations are summarized in Table 2.

The top–down method was applied for the preparation of the formulations, as described in [32].

#### 2.2.3. Dynamic, Static and Electrophoretic Light Scattering Techniques

The physicochemical and morphological properties considered were size (hydrodynamic radius *R_h_*, nm) and size distribution (polydispersity index, PDI) obtained by dynamic light scattering (DLS); ζ-potential (ζ-pot, mV) obtained by electrophoretic light scattering (ELS); *R_g_*/*R_h_* ratio; and *d_f_* by static light scattering (SLS). The *R_h_*, PDI and ζ-pot values were measured in triplicate, and then averaged and reported as a mean ± standard deviation. Statistical analysis was performed by using Student’s *t*-test and multiple comparisons by using one-way ANOVA. *p*-values < 0.05 were considered statistically significant. Measurement details are reported by previous studies [33,36,37,38,39], as well as in the Supplemental Materials.

#### 2.2.4. Cryogenic Transmission Electron Microscopy (Cryo-TEM)

Microscopic illustration of the internal nanostructure was carried out by cryo-TEM for precise visualization of the structures [32,36].

#### 2.2.5. Fluorescence Spectroscopy

Using pyrene as the hydrophobic probe, fluorescence spectroscopy disclosed the internal nanoparticle microenvironment (micropolarity and microfluidity). The intensity ratio of peak 1 to peak 3 of the pyrene (*I*_1_/*I*_3_) serves as a measure of the micropolarity, while the fluorescence intensity ratio *I_E_*/*I_M_* (*I_E_* of pyrene excimer and *I_E_* of pyrene monomer respectively) serves as an index of microfluidity. Larger values indicated increased polarity and fluidity, respectively. The measurements were carried out at two temperatures, 25 °C and 45 °C [36].

#### 2.2.6. Determination of the Drug Entrapment Efficiency (EE)%

Incorporation of 2 mg/mL resveratrol into the prepared nanosystems was attempted; subsequently, the non-entrapped molecules were removed and the entrapped molecules were quantified by UV−Vis spectrophotometry. More technical details may be found in Chountoulesi et al. [32], and in the Supplementary Materials.

## 3. Results and Discussion

### 3.1. Light Scattering Results

TPP-QPDMAEMA-b-PLMA had difficulties in stabilizing the PHYT lipid to nanoparticles, not only alone but also co-assisted by the other polymers, while a stepwise acidification to pH = 5.0 was required to achieve homogenous dispersions. This observation is in contrast to the respective PDMAEMA-b-PLMA-stabilized nanosystems from our previous study [37], where the GMO-based nanosystems were stabilized without acidification. The lipophilicity of the TPP cation of the present polymer likely affects its interactions with the different lipids. The acidification yielding to the ionization of PDMAEMA embraced the hydrophilic character of the copolymer, eventually assisting to stabilize the nanosystem. Afterward, the larger hydrophilic-to-hydrophobic ratio characterizes the amphiphilic stabilizers, and the stringer stabilization is provoked, due to a greater entropic effect [40,41]. Regarding the stabilization process, the block copolymer TPP-QPDMAEMA-b-PLMA was custom synthesized to exhibit a comparable effect to Poloxamer P407, due to its similar hydrophilic–hydrophobic ratio, i.e., 25% PLMA hydrophobic content (Table 1), as the PPO hydrophobic content in Poloxamer P407.

The polymeric guests had similar impact on the physicochemical properties of the formulations (Table 3).

However, we observed higher values of both size and size distribution in comparison to the glyceryl monooleate (GMO)-based respective nanosystems [32], which highlight a different mode of each lipid GMO or PHYT, having a different chemical structure with the polymeric guest. For example, in the case of P407, its hydrophobic block PPO is known to be less attracted from the PHYT lipidic bilayers, because the PPO methyl groups prevent the P407 incorporation within the PHYT backbone and yield only to surface polymer localization onto the nanoparticle, in contrast to GMO cases [42,43], which generate different physicochemical behavior. A similar impact of the PPO block has, in the current cases, the hydrophobic block of PLMA, comprising 25% wt of the copolymer (Table 1). Less pertubation of the hydrophobic block PLMA within the PHYT lipid bilayers and its lower affinity may be responsible for the acidification needed for stabilization; the hydrophilic-block PDMAEMA becomes more ionized and assists more extensively in the stabilization process.

The strong positive ζ-potential in all the formulations (Table 3) reflect the impact of the strong delocalized cation TPP combined with some positively ionized free amino groups of the unquaternized portion of PDMAEMA blocks. Taking into account that the P407, PEO-b-PCL and MPOx copolymers provide negative to neutral particle charge [36,44], we conclude that the TPP-QPDMAEMA-b-PLMA copolymer presence prevails against them. Plus, this strong cationic potential of the PHYT-based systems (>+34 mV, most ζ-potential values larger than + 47 mV) is found to be stronger than the GMO-based systems. This strong positive charge would be helpful for the internalization process by the negatively charged cellular membranes, and could increase the muco-adhesiveness and tissue penetration in cases of mucus delivery routes, and could also be helpful for the encapsulation of nucleic acids, proteins or peptides [45]. Moreover, the observed white nontransparent hue is in agreement with the literature, reflecting 3-D internal conformations [46,47], and a satisfying colloidal stability over time was measured (Figures S2 and S3).

Regarding their ability to incorporate hydrophobic active agents, all the systems were found to successfully incorporate resveratrol in percentages larger than 99% (Table S1), a fact that can be correlated with their exhibiting a compact and extended inner surface structure, due to the presence of PHYT lipid. This is described further in Section 3.2.

#### 3.1.1. Effect of Environmental Parameters

The partial polymer TPP-quaternization synthesis [32] that was carried out left free segments of DMAEMA, suitable for measuring pH response. Taking this into account, it is crucial to test if the resultant nanosystems inherited this property. The tested pH media, apart from the HPLC grade water (pH = 6.0), were the PBS buffer with pH = 7.4, simulating physiological conditions, and citrate buffer with pH = 4.2, simulating the acidic values of endosome lumens, or the pathological conditions, such as the tumor environment [48,49]. For example, the lysosomes and the late endosomes are known to be acidic organelles, exhibiting a pH at 4.0–5.5, due to the presence degradating enzymes and proton pumps (v-ATPases) that maintain this pH range; thus, the response to such conditions could facilitate endosomal escape and protection of the premature degradation of the nanoparticle until the successful content delivery [48,49]. Tumors, in contrast to normal tissues, often exhibit a lower extracellular pH, with some regions reaching below 5. This phenomenon is caused by the tumor’s altered metabolism, particularly increased glycolysis and inefficient mitochondrial oxidative phosphorylation, leading to lactate and proton buildup. That is why pH-responsive nanosystems responding to acidic environments are used for tumor targeting [49].

**Figure 1:**
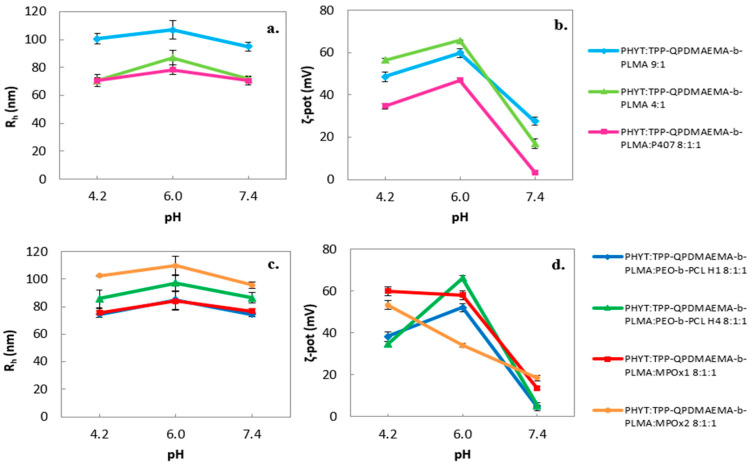
pH effect on the physicochemical behavior of the formulations (size as *R_h_* (nm) (**a**,**c**) and ζ-potential as ζ-pot (mV) (**b**,**d**).

Interestingly, a pH-triggered ζ-potential variation was observed (Figure 1).

In acidic conditions (pH = 4.2), all the nanosystems maintained high positive values of ζ-potential (larger than +30 mV), becoming notably lower in physiological pH. As the literature describes, PDMAEMA is a cationic polyelectrolyte whose tertiary amine groups are fully protonated at acidic pH and becoming less ionized in neutral pH conditions [50,51].

Presumably, this response can be related to the promotion of intracellular accumulation of the nanosystems, with targeting emphasis to pathological acidic tissues and endosomal escape. In detail, the high content of ions eventually floods and ruptures the endosomal membrane and eventually releases the nanoparticle to cytosol, thus being free to interact with the various negatively charged organelles of high targeting interest, like the mitochondria. Moreover, the strong cationic profile of the formulations is favorable in the case of gene or protein or peptide delivery systems [48,49,51].

Regarding the behavior in the presence of serum proteins (fetal bovine serum—FBS medium), most of the nanosystems presented small increases in their size, apart from two exceptions, which showed a slight decrease (Table 4). Thus, we observe that the TPP-quaternized copolymer managed to prevent the nanoparticle disintegration from the albumin, which had taken place in the respective PDMAEMA-b-PLMA-stabilized nanosystems [36,37,44]. Thereby, the present stabilizer, either individually or in combination with the other copolymers, is able to provide stealth PHYT-based nanoparticles. The protein adsorption resistance observed in the present systems likely contributes to stealth properties in vivo, by minimizing opsonization and subsequently preventing uptake by the mononuclear phagocyte system. This can be translated into prolonged circulation time, enhanced bioavailability and improved pharmacokinetics—all of which are critical for effective drug delivery. The ζ-potential values of all the systems exhibited an acute decrease after their interaction with the negatively charged proteins, while the PDI values exhibited an increase, probably due to complexation phenomena with the protein macromolecules.

**Figure 2:**
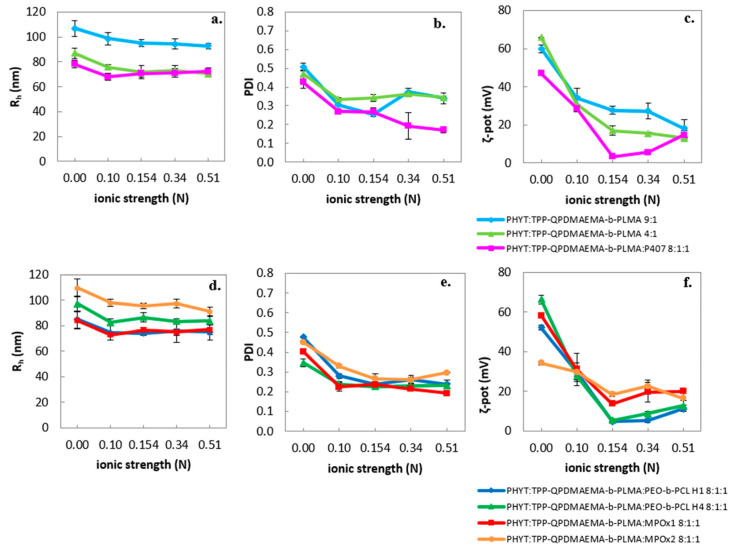
Ionic strength effect on the physicochemical behavior of the formulations (size as *R_h_* (nm) (**a**,**d**), size distribution as PDI (**b**,**e**) and ζ-potential as ζ-pot (mV) (**c**,**f**)).

The ionic strength had an inversely proportional impact on the ζ-potential of the formulations (Figure 2), probably due to cation neutralization by the increased anion content.

#### 3.1.2. Fractal Analysis

Through SLS application, two morphological characteristics were estimated, namely the fractal dimension and the *R_g_*/*R_h_* ratio, reflection shape information (Table 5).

According to the literature, in the case of macromolecular chains, an *R_g_*/*R_h_* of 0.775 reflects a hard uniform sphere, 1.0 indicates vesicles with thin walls and 1.3–1.5 signifies loose conformations in the case of macromolecular chains.

In HPLC-grade water, the systems exhibited different values of the *R_g_*/*R_h_* ratio, around 0.775, implying the prevalence of mostly hard uniform spheres, similar to compact non-lamellar particles and less like vesicle-like morphology, which is observed in liposomal systems [36,38,44]. In an acidic environment with higher temperature, simulating pathological conditions, there were shifts of the *R_g_*/*R_h_* ratio to increased values closer to 1.0 (except for the system containing PEO-b-PCL H4 copolymer), reflecting some morphological re-conformation and disturbance of the nanoassembly shape.

In contrast to the *R_g_*/*R_h_* ratio, the *d_f_* increase in all formulations under the simulated pathological conditions confirms that the stimuli-responsiveness was also inherited by the morphological, internal conformation. The lower pH increases the number of ionized amine groups of PDMAEMA, leading to less Gaussian-curved assemblies and thus altered *d_f_* [38]. The correlation between ion content within the liquid crystalline nanosystems and Gaussian curvature has also been verified by other studies in the literature [25,52].

#### 3.1.3. Temperature Effect

**Figure 3:**
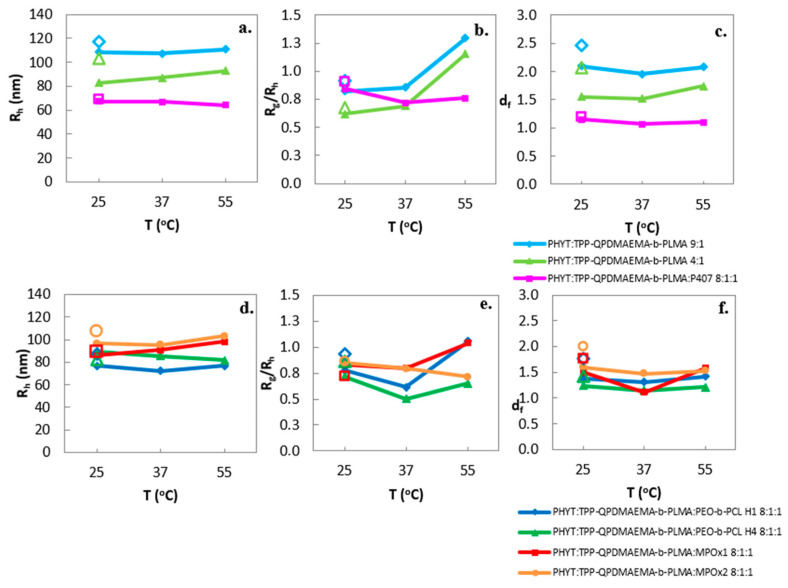
Temperature effect on the physicochemical and morphological behavior of the formulations (size as *R_h_* (nm) (**a**,**d**), *R_g_*/*R_h_* (**b**,**e**) and fractal dimension (**c**,**f**)). Empty markers indicate measurements taken after the cooling cycle.

The effect of rising temperature and subsequent cooling was monitored for the physicochemical properties of the formulations, with no significant variations in hydrodynamic radius and PDI between the heating and cooling cycles (Figure 3 and Figure S5).

Concerning the morphological parameters, the *R_g_*/*R_h_* ratio in most of the nanosystems increased after reaching 37 °C, indicating a morphologically reversible re-arrangement to more loose nanoassemblies, similarly to the classical case with only the P407 stabilizer [36]. Moreover, Muller et al. [53] also described a similar reversible transformation, i.e., a heated inverse micellar solution (*L*_2_ phase) above 50 °C, which was cooled to *Pn3m* symmetry PHYT cubosomes. Contrariwise, no significant alterations of *d_f_* were observed.

### 3.2. Cryo-TEM Results

**Figure 4:**
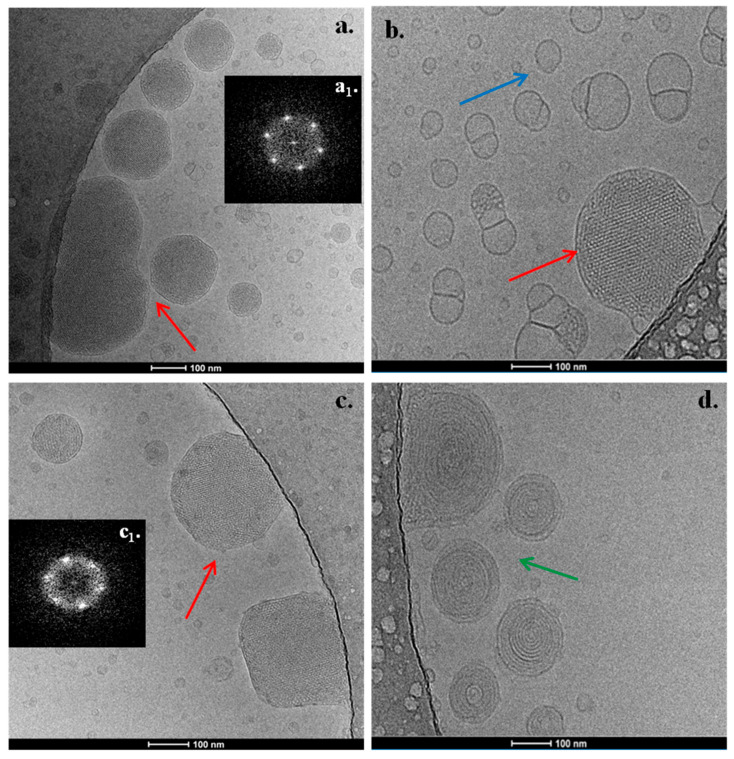
Cryo-TEM images from (**a**) PHYT:TPP-QPDMAEMA-b-PLMA 9:1, (**b**) PHYT:TPP-QPDMAEMA-b-PLMA:P407 8:1:1, (**c**,**d**) PHYT:TPP-QPDMAEMA-b-PLMA 4:1 formulations. The FFT patterns (figure **a_1_**, **c_1_**) indicate *Pn3m* cubic symmetry.

The PHYT-based nanosystems exhibited a variety of morphological features. The PHYT:TPP-QPDMAEMA-b-PLMA 9:1 nanosystem presented nanoparticles with striated, regularly ordered, inner structure, as illustrated in Figure 4a (red arrow). The corresponding FFT patterns (insets, Figure 4(a**_1_**)) indicate *Pn3m* cubic symmetry [54].

A cubic phase with the *Pn3m* crystallographic space group is typically found in PHYT systems, based on reports in the literature [25]. According to Dong et al. [42], P407, being surface absorbed and not incorporated within the PHYT bilayer, prevents the phenomenon of the double-diamond type (*Pn3m*) to primitive type (*Im3m*) phase transition, as in the case of GMO cubosomes. We assume that this phenomenon also takes place in the case of systems containing the TPP-QPDMAEMA-b-PLMA copolymer. Apart from the compact nanoparticles, empty vesicles and micelles were also observed.

The addition of P407 as a second stabilizer to the formulation (Figure 4) led to the coexistence of regularly ordered liquid crystalline nanoparticles (red arrow), along with small, spherical vesicles with no inner structure (blue arrow). We should mention that the observed morphological behavior differs from the respective PHYT:P407 nanosystems that we investigated in our previous study [36], where there was an absence of empty vesicles, highlighting the effect of the TPP-QPDMAEMA-b-PLMA copolymer on the morphology of the nanostructure.

The increase in TPP-QPDMAEMA-b-PLMA copolymer to the 4:1 ratio resulted in the formation of two populations of nanostructures, while there was an appearance of spherical multilamellar vesicles (Figure 4d, green arrow), apart from the presence of regularly ordered nanoparticles (Figure 4c, red arrow) of *Pn3m* symmetry. Generally, the nanoparticles indicated by a green arrow tend more toward hexosome morphologies [54,55,56,57,58,59,60].

**Figure 5:**
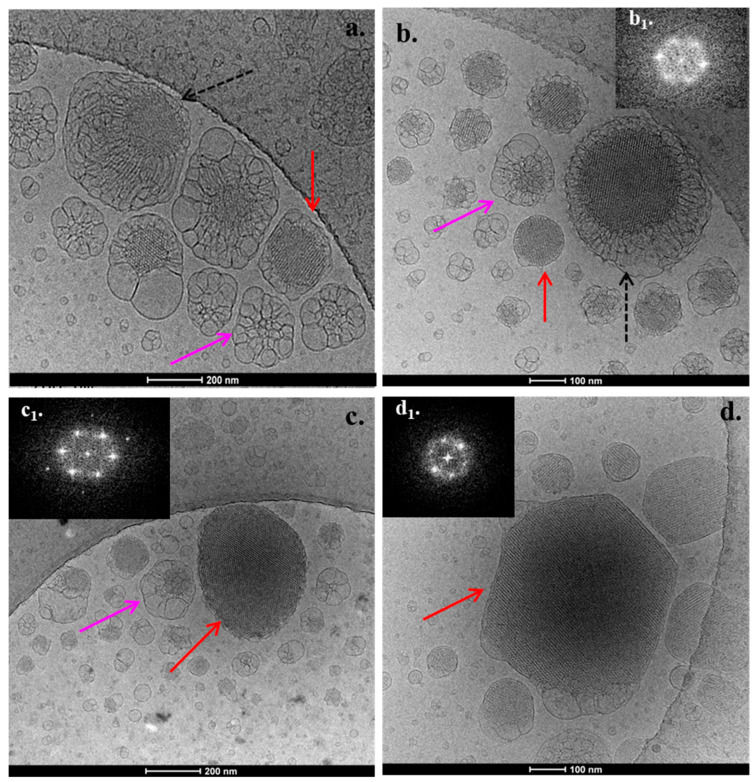
Cryo-TEM images from (**a**) PHYT:TPP-QPDMAEMA-b-PLMA:PEO-b-PCL H1 8:1:1, (**b**) PHYT:TPP-QPDMAEMA-b-PLMA:PEO-b-PCL H4 8:1:1, (**c**) PHYT:TPP-QPDMAEMA-b-PLMA:MPOx1 8:1:1, (**d**) PHYT:TPP-QPDMAEMA-b-PLMA:MPOx2 8:1:1 formulations. The FFT patterns (figure **b_1_**–**d_1_**) indicate *Pn3m* cubic symmetry.

The addition of the PEO-b-PCL copolymers differentiated the layout of regularly ordered structures. In the formulation with PEO-b-PCL H1, compact liquid crystalline nanoparticles of highly ordered inner structure are present (Figure 5a, red arrow), likely exhibiting a *Pn3m* space group symmetry [61], as was assessed by the FFT. However, the special feature of systems that contain PEO-b-PCL is the fact that some of the illustrated ordered nanoparticles are embedded in a plethora of large intersecting lamellas (Figure 5a, black discontinuous arrows). Another interesting observation is some bulgy nanoparticles related to fusion phenomena, either containing disordered nanostructures in their center or not (Figure 5a, pink arrow).

In the formulation with PEO-b-PCL H4, there were the same three aforementioned categories of 3D morphologies (Figure 5b, red, black and pink arrows) along with some multilamellar nanoparticles presenting different stages of disorganization. We should mention that these disorganized objects along with the bulgy nanoparticles, indicated by the pink arrows, possibly correlate with the acidification that was used during the preparation of the PHYT-based nanosystems, as was found in our previous study [32].

The insertion of MPOx1 gradient copolymer resulted in the formation of similar nanostructures, as in the case of PEO-b-PCL-containing nanosystems. In detail, liquid crystalline nanoparticles with a highly ordered inner structure and likely space group symmetry *Pn3m* were observed (Figure 5c, red arrow), along with ordered nanoparticles coming out from a plethora of large intersecting lamellas, together with some bulgy nanoparticles (Figure 5c, pink arrow).

Finally, the insertion of the MPOx2 gradient copolymer resulted in the formation of liquid crystalline nanoparticles with a highly ordered inner structure and with likely space group symmetry *Pn3m* (Figure 5d, red arrow), but not surrounded by lamellas on their surface. Moreover, a population of bulgy nanoparticles (Figure 5d, pink arrow) from fused vesicular structures is also present.

Taking into account the morphological behavior in the case of the GMO lipid in our previous study [32], the replacement with the PHYT lipid led to a reduction in the dominant number of empty vesicles and to the formation of larger, compact structures with organized symmetries, a fact that was also observed in the model systems containing only GMO/PHYT and P407 [36]. This observation indicates the crucial role of lipid selection to the behavior of the system and the entire formulation process. In the present study, the lipid played a predominant role in the morphology of the nanoparticles that formed, while the same categories of nanostructures were found in the majority of the polymers that were used.

As long as there are different polymeric architecture effects, the systems comprising MPOx gradient copolymers as stabilizers reflect a possibly more packed lipid bilayer structure, yielding to more compact nanoparticulate morphologies, compared to the ones comprising the di-block copolymers, TPP-QPDMAEMA-b-PLMA alone or with the di-block copolymers PEO-b-PCL/P407, while the systems comprising MPOx gradient copolymers also exhibit darker nanoparticle motifs, show less fusion phenomena and reveal more “bare” nanoparticles, without being embedded in intersecting lamellas and sponges (Figure 5c,d). The observed phenomena can be attributed to contrasting copolymer architecture between the gradient and block copolymers that yield to a different mode of interaction with the lipids, and thus a different mode of localization within the lipid membrane. In more detail, the biomolecular sculpture of MPOx gradient copolymer is known to have several entry and exit points in the lipid membrane, in contrast to amphiphilic di-block copolymers, wherein there is only one point with the hydrophobic block to become anchored/incorporated into the lipid bilayer and the hydrophilic block stretching out from the membrane [62]. We assume that this gradient type of polymer arrangement generates the more compact morphologies that were observed.

### 3.3. Fluorescence Spectroscopy Results

All of the nanosystems exhibit similar values of *I*_1_/*I*_3_ (up to 0.84) (Table 6), regardless of the pH, temperature or type and content of stabilizer.

We should mention that the observed micropolarity values are lower than the respective GMO nanosystems [32], which were found to be around 1.04, thus reflecting less polar bilayers/domains in the case of the PHYT systems. This agrees with the results from previous studies, where P407 was used as the stabilizer in both GMO and PHYT nanosystems [36]. The different lipidic chemical structures (absence of double bond in PHYT) differentiate the insertion mode of the probe and arrangement within the structures. The literature describes different interactions that take place between the lipid and stabilizer, which depends on the lipid’s chemical structure. For example, a different entrance rate for the PPO block occurs in P407 (surface localized in the PHYT formulations and inner perturbation in the GMO formulations). This perturbation in the GMO formulations, which also provokes the *Pn3m* to *Im3m* transition, enables water absorbance from the environment and eventually more polar bilayers [63], contrary to PHYT formulations that are characterized by more hydrophobic profiles [42,64]. These interactions are probably also provoked by the TPP-QPDMAEMA-b-PLMA polymer.

By combining the results of cryo-TEM and fluorescence spectroscopy, which present the dominant existence of *Pn3m* symmetry and the absence of the *Im3m* transition, we can assess some conclusions regarding the stability of the inner structure of the PHYT systems. According to the literature, in GMO-based systems, the gradual increase in the pertubating polymeric stabilizer results from the *Pn3m* to *Im3m* transition, which is a less negatively curved phase, to the loss of the inner organized structure, in the manner of a polymer concentration analogue, due to the already known swelling of the phase that results in a phase transition from cubic to lamellar [57,65]. In the present newly proposed PHYT-copolymer systems, the persistence of *Pn3m* symmetry in every copolymer concentration and combination indicates a higher stability of the inner structure of the nanoparticle compared to the GMO-respective nanosystems of [20]. This greater stability can maintain the specifications, like particle size, shape and a stable morphology, and prevent undesired content leakages. Cubic phases of the *Pn3m* type, as in the present systems, exhibit four aqueous channels (meeting at a tetrahedral angle), which is less than for other morphologies like the *Im3m* type, thus yielding to more prolonged release profiles [66].

Another significant observation is the decrease in microfluidity (*I_E_*/*I_M_*) at 45 °C at both pH values. The temperature-responsive shrinkage of the PDMAEMA polymeric corona in temperatures higher than its LCST perhaps makes the bilayer more fragile and less viscous, thus accessible to pyrene, increasing the microfluidity. A similar trend has also been found in the GMO-based respective systems, which was correlated with the increased drug release under these conditions [32]. In other systems with the thermoresponsive polymer that includes poly(N-isopropylacrylamide) (PNIPAM), the increased temperature also yielded to dehydration and a decrease in the water–lipid polar-head affinity, thus enhancing the release of a hydrophilic water cargo [65]. This observed response to an increase in temperature can prove to be very useful toward the facilitation of the temperature-induced release of entrapped hydrophobic drugs from the membranes.

## 4. Conclusions

Non-lamellar liquid crystalline nanosystems were formulated from PHYT lipid and the stimuli-responsive polymer TPP-QPDMAEMA-b-PLMA, assisted by different types of other copolymers, either block copolymers Poloxamer P407 and PEO-b-PCL, or gradient copolymers MPOx. All formulations presented a strong cationic profile, along with a pH-induced ζ-potential conversion in acidic conditions that can be further utilized for intracellular targeting applications. In addition, the different polymeric combinations played a crucial role in the morphology of the nanosystems, wherein a prevailing population of compact structures with a high internal organization was found, probably due to the effect of the phytantriol lipid. This newly proposed PHYT lipid–copolymer combination yielded to a new morphologically combined nanoparticulate structure of a well-defined liquid crystalline nanoparticle of *Pn3m* symmetry, being embedded in a plethora of large intersecting lamellas. Moreover, this newly proposed PHYT lipid–copolymer combination provoked a decrease in empty vesicle population within the dispersion, and an increase in the organized structures. This result embraces all the advantages of the liquid crystalline nanostructures versus the empty ones, like the enhanced loading capacity, as this was also proven by the results. Indeed, the studied nanosystems were able to incorporate high percentages of hydrophobic active molecules. Further, a strong cationic potential was found (> +34 mV, most ζ-potential values larger than +47 mV), stronger than that recorded for GMO-systems, while the proposed copolymers retain the stable double-diamond symmetry of the PHYT cubosomes. In conclusion, the proposed liquid crystalline formulations are promising drug delivery nanocarrier candidates, and especially for the cases in which cationic charge is needed.

## Figures and Tables

**Table 1:** Molecular characteristics of the TPP-QPDMAEMA-b-PLMA, PEO-b-PCL and MPOx copolymers used in this study.

Copolymer	M_w_ ^a^	M_w_/M_n_ ^a^	%wt Hydrophobic Component ^b^	Reference with Copolymer Synthesis
TPP-QPDMAEMA-b-PLMA	8491	1.17	%wt PLMA 25	[32,35]
PEO-b-PCL H1	5900	1.04	%wt PCL 15	[33]
PEO-b-PCL H4	7100	1.18	%wt PCL 30	[33]
MPOx1	5200	1.14	%wt PhOx 28	[34]
MPOx2	3200	1.15	%wt PhOx 10	[34]

^a^ by SEC in CHCl_3_ using polystyrene standards. ^b^ by ^1^H-NMR in CDCl_3_.

**Table 2:** Content description of the formulations.

Sample	Lipid Polymer Weight Ratio	Stabilizer Copolymer Content	Total Copolymers Percentage
PHYT:TPP-QPDMAEMA-b-PLMA	9:1	TPP-QPDMAEMA-b-PLMA	10%
PHYT:TPP-QPDMAEMA-b-PLMA	4:1	TPP-QPDMAEMA-b-PLMA	20%
PHYT:TPP-QPDMAEMA-b-PLMA:P407	8:1:1	TPP-QPDMAEMA-b-PLMA and Poloxamer P407	20%
PHYT:TPP-QPDMAEMA-b-PLMA:PEO-b-PCL H1	8:1:1	TPP-QPDMAEMA-b-PLMA and PEO-b-PCL H1	20%
PHYT:TPP-QPDMAEMA-b-PLMA:PEO-b-PCL H4	8:1:1	TPP-QPDMAEMA-b-PLMA and PEO-b-PCL H4	20%
PHYT:TPP-QPDMAEMA-b-PLMA:MPOx1	8:1:1	TPP-QPDMAEMA-b-PLMA and MPOx1	20%
PHYT:TPP-QPDMAEMA-b-PLMA:MPOx2	8:1:1	TPP-QPDMAEMA-b-PLMA and MPOx2	20%

**Table 3:** Physicochemical behavior (size as *R_h_* (nm), size distribution as PDI and ζ-potential as ζ-pot (mV)) on the day of preparation (25 °C).

Sample	Weight Ratio	*R_h_* (nm)	PDI	ζ-Pot (mV)
PHYT:TPP-QPDMAEMA-b-PLMA	9:1	106.9 ± 6.4	0.508 ± 0.019	59.9 ± 2.1
PHYT:TPP-QPDMAEMA-b-PLMA	4:1	87.1 ± 5.4	0.471 ± 0.016	65.8 ± 0.1
PHYT:TPP-QPDMAEMA-b-PLMA:P407	8:1:1	78.4 ± 3.4	0.426 ± 0.032	47.0 ± 1.2
PHYT:TPP-QPDMAEMA-b-PLMA:PEO-b-PCL H1	8:1:1	84.8 ± 6.9	0.476 ± 0.003	52.1 ± 1.1
PHYT:TPP-QPDMAEMA-b-PLMA:PEO-b-PCL H4	8:1:1	97.3 ± 6.0	0.347 ± 0.021	66.2 ± 2.1
PHYT:TPP-QPDMAEMA-b-PLMA:MPOx1	8:1:1	84.2 ± 6.7	0.404 ± 0.003	58.2 ± 0.5
PHYT:TPP-QPDMAEMA-b-PLMA:MPOx2	8:1:1	109.7 ± 7.1	0.451 ± 0.007	34.2 ± 1.0

**Table 4:** Serum protein effect on the physicochemical behavior of the formulations (size as *R_h_* (nm), size distribution as PDI and ζ-potential as ζ-pot (mV)).

Sample	Weight Ratio	Medium	*R_h_* (nm)	PDI	ζ-Pot (mV)
PHYT:TPP-QPDMAEMA-b-PLMA	9:1	Water *	106.9 ± 6.4	0.508 ± 0.019	59.9 ± 2.1
		FBS	91.7 ± 3.0	1.000 ± 0.000	−6.9 ± 0.1
PHYT:TPP-QPDMAEMA-b-PLMA	4:1	Water *	87.1 ± 5.4	0.471 ± 0.016	65.8 ± 0.1
		FBS	117.9 ± 1.9	1.000 ± 0.000	13.7 ± 1.0
PHYT:TPP-QPDMAEMA-b-PLMA:P407	8:1:1	Water *	78.4 ± 3.4	0.426 ± 0.032	47.0 ± 1.2
		FBS	82.6 ± 2.1	1.000 ± 0.000	2.1 ± 0.1
PHYT:TPP-QPDMAEMA-b-PLMA:PEO-b-PCL H1	8:1:1	Water *	84.8 ± 6.9	0.476 ± 0.003	52.1 ± 1.1
		FBS	83.9 ± 1.8	0.929 ± 0.006	−4.2 ± 0.2
PHYT:TPP-QPDMAEMA-b-PLMA:PEO-b-PCL H4	8:1:1	Water *	97.3 ± 6.0	0.347 ± 0.021	66.2 ± 2.1
		FBS	99.1 ± 1.2	1.000 ± 0.000	3.5 ± 0.2
PHYT:TPP-QPDMAEMA-b-PLMA:MPOx1	8:1:1	Water *	84.2 ± 6.7	0.404 ± 0.003	58.2 ± 0.5
		FBS	110.1 ± 19.6	0.894 ± 0.005	−4.2 ± 0.2
PHYT:TPP-QPDMAEMA-b-PLMA:MPOx2	8:1:1	Water *	109.7 ± 7.1	0.451 ± 0.007	34.2 ± 1.0
		FBS	115.0 ± 5.1	1.000 ± 0.000	2.1 ± 0.3

* HPLC-grade water.

**Table 5:** *R_g_*/*R_h_* and fractal dimension *d_f_* under different pH and temperature conditions.

Sample	Weight Ratio	pH	T (°C)	*R_g_*/*R_h_*	*d_f_*
PHYT:TPP-QPDMAEMA-b-PLMA	9:1	4.2	25	0.95	1.96
		6.0	42	0.82	2.10
PHYT:TPP-QPDMAEMA-b-PLMA	4:1	4.2	25	0.79	1.59
		6.0	42	0.62	1.55
PHYT:TPP-QPDMAEMA-b-PLMA:P407	8:1:1	4.2	25	0.97	1.43
		6.0	42	0.84	1.15
PHYT:TPP-QPDMAEMA-b-PLMA:PEO-b-PCL H1	8:1:1	4.2	25	0.90	1.51
		6.0	42	0.77	1.37
PHYT:TPP-QPDMAEMA-b-PLMA:PEO-b-PCL H4	8:1:1	4.2	25	0.70	1.30
		6.0	42	0.72	1.24
PHYT:TPP-QPDMAEMA-b-PLMA:MPOx1	8:1:1	4.2	25	0.86	1.79
		6.0	42	0.83	1.50
PHYT:TPP-QPDMAEMA-b-PLMA:MPOx2	8:1:1	4.2	25	0.86	1.83
		6.0	42	0.85	1.60

**Table 6:** *I*_1_/*I*_3_ (as micropolarity) and *I_E_*/*I_M_* (as microfluidity).

Sample	Weight Ratio	T (°C)	pH = 6.0		pH = 4.2	
			*I*_1_/*I*_3_	*Ι*_Ε_/*Ι*_Μ_	*I*_1_/*I*_3_	*Ι_Ε_*/*Ι_Μ_*
PHYT:TPP-QPDMAEMA-b-PLMA	9:1	25	0.79	0.09	0.79	0.09
		45	0.79	0.14	0.79	0.14
PHYT:TPP-QPDMAEMA-b-PLMA	4:1	25	0.98	0.05	0.98	0.05
		45	0.81	0.09	0.81	0.09
PHYT:TPP-QPDMAEMA-b-PLMA:P407	8:1:1	25	0.81	0.06	0.81	0.06
		45	0.81	0.10	0.81	0.10
PHYT:TPP-QPDMAEMA-b-PLMA:PEO-b-PCL H1	8:1:1	25	0.82	0.06	0.82	0.06
		45	0.80	0.11	0.80	0.11
PHYT:TPP-QPDMAEMA-b-PLMA:PEO-b-PCL H4	8:1:1	25	0.87	0.06	0.87	0.06
		45	0.84	0.11	0.84	0.11
PHYT:TPP-QPDMAEMA-b-PLMA:MPOx1	8:1:1	25	0.81	0.06	0.81	0.06
		45	0.79	0.12	0.79	0.12
PHYT:TPP-QPDMAEMA-b-PLMA:MPOx2	8:1:1	25	0.81	0.07	0.81	0.07
		45	0.79	0.12	0.79	0.12

## Data Availability

Data is contained within the article or Supplementary Materials.

## Supplementary Materials

The following supporting information can be downloaded at https://www.mdpi.com/article/10.3390/pharmaceutics17091199/s1, Figure S1: Chemical structures of (**a**) PHYT lipid, (**b**) TPP-QPDMAEMA-b-PLMA block copolymer, (**c**) P407 (PEO_98_-PPO_67_-PEO_98_) copolymer, (**d**) PEO-b-PCL block copolymers and (**e**) MPOx gradient copolymers employed in this study.; Figure S2: Stability assessment of the hydrodynamic radius (*R_h_*, nm) of the prepared nanosystems over time.; Figure S3: Stability assessment of the polydispersity index (PDI) of the prepared nanosystems over time.; Figure S4: Polydispersity index (PDI) of the prepared nanosystems in aqueous media with different pH. Figure S5: Polydispersity index (PDI) of the prepared nanosystems vs. temperature.; Table S1: Encapsulation efficiency (EE%) of the resveratrol.

## Abbreviations

PDMAEMA-b-PLMA	poly(2-(dimethylamino)ethyl methacrylate)-b-poly(lauryl methacrylate)
TPP	tri-phenyl-phosphine cation
PEO-b-PCL	poly(ethylene oxide)-block-poly(ε-caprolactone)
MPOx	poly(2-methyl-2-oxazoline)-grad-poly(2-phenyl-2-oxazoline)
P407	Poloxamer P407/Pluronic F-127
PPO	Poly(propylene oxide)
PEO	Poly(ethylene oxide)
GMO	Glyceryl monooleate
PHYT	Phytantriol
R_h_	Hydrodynamic radius
R_g_	Radius of gyration
PDI	Polydispersity index
ζ-pot	ζ-potential
d_f_	Fractal dimension
Cryo-TEM	Cryogenic transmission electron microscopy
DLS	Dynamic light scattering
ELS	Electrophoretic light scattering
SLS	Static light scattering
FBS	Fetal bovine serum
PBS	Phosphate buffered saline

## References

[1] Zhai J., Fong C., Tran N., Drummond C.J. (2019). Non-Lamellar Lyotropic Liquid Crystalline Lipid Nanoparticles for the Next Generation of Nanomedicine. ACS Nano.

[2] Barriga H.M.G., Holme M.N., Stevens M.M. (2019). Cubosomes: The Next Generation of Smart Lipid Nanoparticles?. Angew. Chem. Int. Ed. Engl..

[3] Fornasier M., Murgia S. (2023). Non-Lamellar Lipid Liquid Crystalline Nanoparticles: A Smart Platform for Nanomedicine Applications. Front. Soft Matter.

[4] Govindan I., Paul A., Rama A., Kailas A.A., Abutwaibe K.A., Annadurai T., Naha A. (2025). Mesogenic Architectures for Advanced Drug Delivery: Interrogating Lyotropic and Thermotropic Liquid Crystals. AAPS PharmSciTech.

[5] Varghese R., Salvi S., Sood P., Kulkarni B., Kumar D. (2022). Cubosomes in Cancer Drug Delivery: A Review. Colloid Interface Sci. Commun..

[6] Oliveira C., Ferreira C.J.O., Sousa M., Paris J.L., Gaspar R., Silva B.F.B., Teixeira J.A., Ferreira-Santos P., Botelho C.M. (2022). A Versatile Nanocarrier—Cubosomes, Characterization, and Applications. Nanomaterials.

[7] Garg S.S., Vyas A., Arivarasan V.K., Gupta J. (2024). Cubosomes: An Emerging Nanodrug Delivery Platform for Anti-Diabetic Medications. J. Drug Deliv. Sci. Technol..

[8] Khoshdooz S., Khoshdooz P., Bonyad R., Bonyad A., Sheidaei S., Nosrati R. (2025). Cubosomes-Based Hydrogels; A Promising Advancement for Drug Delivery. Int. J. Pharm..

[9] Palma A.S., Casadei B.R., Lotierzo M.C., Dias de Castro R., Barbosa L.R.S. (2023). A Short Review on the Applicability and Use of Cubosomes as Nanocarriers. Biophys. Rev..

[10] Sivadasan D., Sultan M.H., Alqahtani S.S., Javed S. (2023). Cubosomes in Drug Delivery—A Comprehensive Review on Its Structural Components, Preparation Techniques and Therapeutic Applications. Biomedicines.

[11] Nath A.G., Dubey P., Kumar A., Vaiphei K.K., Rosenholm J.M., Bansal K.K., Gulbake A. (2024). Recent Advances in the Use of Cubosomes as Drug Carriers with Special Emphasis on Topical Applications. J. Lipids.

[12] Shetty S., Shetty S. (2023). Cubosome-Based Cosmeceuticals: A Breakthrough in Skincare. Drug Discov. Today.

[13] Madheswaran T., Kandasamy M., Bose R.J., Karuppagounder V. (2019). Current Potential and Challenges in the Advances of Liquid Crystalline Nanoparticles as Drug Delivery Systems. Drug Discov. Today.

[14] Priya S., Desai V.M., Singhvi G. (2024). Tailoring Lyotropic Liquid Crystals for Skin Barrier Penetration: Exploring Composition and Structure–Function Relationships. Appl. Phys. Rev..

[15] Seo Y., Lim H., Park H., Yu J., An J., Yoo H.Y., Lee T. (2023). Recent Progress of Lipid Nanoparticles-Based Lipophilic Drug Delivery: Focus on Surface Modifications. Pharmaceutics.

[16] Guedes M.D.V., Marques M.S., Berlitz S.J., Facure M.H.M., Correa D.S., Steffens C., Contri R.V., Külkamp-Guerreiro I.C. (2023). Lamivudine and Zidovudine-Loaded Nanostructures: Green Chemistry Preparation for Pediatric Oral Administration. Nanomaterials.

[17] Blanco-Fernández G., Blanco-Fernandez B., Fernández-Ferreiro A., Otero-Espinar F.J. (2023). Lipidic Lyotropic Liquid Crystals: Insights on Biomedical Applications. Adv. Colloid Interface Sci..

[18] Adwan S., Qasmieh M., Al-Akayleh F., Ali Agha A.S.A. (2024). Recent Advances in Ocular Drug Delivery: Insights into Lyotropic Liquid Crystals. Pharmaceuticals.

[19] Blanco-Fernandez G., Blanco-Fernandez B., Fernández-Ferreiro A., Otero-Espinar F. (2023). Bringing Lipidic Lyotropic Liquid Crystal Technology into Biomedicine. Trends Pharmacol. Sci..

[20] Baldha R., Chakraborthy G.S., Rathod S. (2025). Current Status and Future Prospects of Lyotropic Liquid Crystals as a Nanocarrier Delivery System for the Treatment of Cancer. AAPS PharmSciTech.

[21] Chavda V.P., Dyawanapelly S., Dawre S., Ferreira-Faria I., Bezbaruah R., Rani Gogoi N., Kolimi P., Dave D.J., Paiva-Santos A.C., Vora L.K. (2023). Lyotropic Liquid Crystalline Phases: Drug Delivery and Biomedical Applications. Int. J. Pharm..

[22] Tarsitano M., Mancuso A., Cristiano M.C., Urbanek K., Torella D., Paolino D. (2023). Perspective Use of Bio-Adhesive Liquid Crystals as Ophthalmic Drug Delivery Systems. Sci. Rep..

[23] Gawarkar-Patil P., Mahajan B., Pawar A., Dhapte-Pawar V. (2024). Cubosomes: Evolving Platform for Intranasal Drug Delivery of Neurotherapeutics. Futur. J. Pharm. Sci..

[24] Kulkarni C.V., Wachter W., Iglesias-Salto G., Engelskirchen S., Ahualli S. (2011). Monoolein: A Magic Lipid?. Phys. Chem. Chem. Phys..

[25] El Mohamad M., Han Q., Clulow A.J., Cao C., Safdar A., Stenzel M., Drummond C.J., Greaves T.L., Zhai J. (2024). Regulating the Structural Polymorphism and Protein Corona Composition of Phytantriol-Based Lipid Nanoparticles Using Choline Ionic Liquids. J. Colloid Interface Sci..

[26] Lima L.A., Moura E.E.L.d., Fraga S.F., Contri R.V., Külkamp-Guerreiro I.C. (2025). Development of Nifedipine Phytantriol-Based Cubosomes and In Vitro Simulation of Administration Through Pediatric Feeding Tubes. Pharmaceutics.

[27] Astolfi P., Giorgini E., Adamo F.C., Vita F., Logrippo S., Francescangeli O., Pisani M. (2019). Effects of a Cationic Surfactant Incorporation in Phytantriol Bulk Cubic Phases and Dispersions Loaded with the Anticancer Drug 5-Fluorouracil. J. Mol. Liq..

[28] Villalva D.G., França C.G., Loh W. (2022). Characterization of Cubosomes Immobilized in Hydrogels of Hyaluronic Acid and Their Use for Diclofenac Controlled Delivery. Colloids Surf. B Biointerfaces.

[29] Rajesh S., Zhai J., Drummond C.J., Tran N. (2022). Novel pH-Responsive Cubosome and Hexosome Lipid Nanocarriers of SN-38 Are Prospective for Cancer Therapy. Pharmaceutics.

[30] Prajapati R., Gontsarik M., Yaghmur A., Salentinig S. (2019). pH-Responsive Nano-Self-Assemblies of the Anticancer Drug 2-Hydroxyoleic Acid. Langmuir.

[31] Mathews P.D., Mertins O., Angelov B., Angelova A. (2022). Cubosomal Lipid Nanoassemblies with pH-Sensitive Shells Created by Biopolymer Complexes: A Synchrotron SAXS Study. J. Colloid Interface Sci..

[32] Chountoulesi M., Perinelli D.R., Forys A., Chrysostomou V., Kaminari A., Bonacucina G., Trzebicka B., Pispas S., Demetzos C. (2023). Development of Stimuli-Responsive Lyotropic Liquid Crystalline Nanoparticles Targeting Lysosomes: Physicochemical, Morphological and Drug Release Studies. Int. J. Pharm..

[33] Pippa N., Kaditi E., Pispas S., Demetzos C. (2013). PEO-b-PCL–DPPC Chimeric Nanocarriers: Self-Assembly Aspects in Aqueous and Biological Media and Drug Incorporation. Soft Matter.

[34] Milonaki Y., Kaditi E., Pispas S., Demetzos C. (2012). Amphiphilic Gradient Copolymers of 2-Methyl- and 2-Phenyl-2-Oxazoline: Self-Organization in Aqueous Media and Drug Encapsulation. J. Polym. Sci. A Polym. Chem..

[35] Chrysostomou V., Pispas S. (2018). Stimuli-Responsive Amphiphilic PDMAEMA-*b*-PLMA Copolymers and Their Cationic and Zwitterionic Analogs. J. Polym. Sci. A Polym. Chem..

[36] Chountoulesi M., Pippa N., Pispas S., Chrysina E.D., Forys A., Trzebicka B., Demetzos C. (2018). Cubic Lyotropic Liquid Crystals as Drug Delivery Carriers: Physicochemical and Morphological Studies. Int. J. Pharm..

[37] Chountoulesi M., Pippa N., Chrysostomou V., Pispas S., Chrysina E.D., Forys A., Otulakowski L., Trzebicka B., Demetzos C. (2019). Stimuli-Responsive Lyotropic Liquid Crystalline Nanosystems with Incorporated Poly(2-Dimethylamino Ethyl Methacrylate)-b-Poly(Lauryl Methacrylate) Amphiphilic Block Copolymer. Polymers.

[38] Chountoulesi M., Perinelli D.R., Pippa N., Chrysostomou V., Forys A., Otulakowski L., Bonacucina G., Trzebicka B., Pispas S., Demetzos C. (2020). Physicochemical, Morphological and Thermal Evaluation of Lyotropic Lipidic Liquid Crystalline Nanoparticles: The Effect of Stimuli-Responsive Polymeric Stabilizer. Colloids Surf. A Physicochem. Eng. Asp..

[39] Pippa N., Pispas S., Demetzos C. (2012). The Delineation of the Morphology of Charged Liposomal Vectors via a Fractal Analysis in Aqueous and Biological Media: Physicochemical and Self-Assembly Studies. Int. J. Pharm..

[40] Chong J.Y.T., Mulet X., Waddington L.J., Boyd B.J., Drummond C.J. (2011). Steric Stabilisation of Self-Assembled Cubic Lyotropic Liquid Crystalline Nanoparticles: High Throughput Evaluation of Triblock Polyethylene Oxide–Polypropylene Oxide–Polyethylene Oxide Copolymers. Soft Matter.

[41] Chong J.Y.T., Mulet X., Waddington L.J., Boyd B.J., Drummond C.J. (2012). High-Throughput Discovery of Novel Steric Stabilizers for Cubic Lyotropic Liquid Crystal Nanoparticle Dispersions. Langmuir.

[42] Dong Y.D., Larson I., Hanley T., Boyd B.J. (2006). Bulk and Dispersed Aqueous Phase Behavior of Phytantriol: Effect of Vitamin E Acetate and F127 Polymer on Liquid Crystal Nanostructure. Langmuir.

[43] Rizwan S.B., Assmus D., Boehnke A., Hanley T., Boyd B.J., Rades T., Hook S. (2011). Preparation of Phytantriol Cubosomes by Solvent Precursor Dilution for the Delivery of Protein Vaccines. Eur. J. Pharm. Biopharm..

[44] Chountoulesi M., Perinelli D.R., Forys A., Bonacucina G., Trzebicka B., Pispas S., Demetzos C. (2021). Liquid Crystalline Nanoparticles for Drug Delivery: The Role of Gradient and Block Copolymers on the Morphology, Internal Organisation and Release Profile. Eur. J. Pharm. Biopharm..

[45] Shawky S., Makled S., Awaad A., Boraie N. (2022). Quercetin Loaded Cationic Solid Lipid Nanoparticles in a Mucoadhesive In Situ Gel—A Novel Intravesical Therapy Tackling Bladder Cancer. Pharmaceutics.

[46] Akhlaghi S.P., Ribeiro I.R., Boyd B.J., Loh W. (2016). Impact of Preparation Method and Variables on the Internal Structure, Morphology, and Presence of Liposomes in Phytantriol–Pluronic^®^ F127 Cubosomes. Colloids Surf. B Biointerfaces.

[47] Liu Q., Dong Y.D., Hanley T.L., Boyd B.J. (2013). Sensitivity of Nanostructure in Charged Cubosomes to Phase Changes Triggered by Ionic Species in Solution. Langmuir.

[48] Kim K., Chen W.C.W., Heo Y., Wang Y. (2016). Polycations and Their Biomedical Applications. Prog. Polym. Sci..

[49] Kanamala M., Wilson W.R., Yang M., Palmer B.D., Wu Z. (2016). Mechanisms and Biomaterials in pH-Responsive Tumour Targeted Drug Delivery: A Review. Biomaterials.

[50] Sprouse D., Jiang Y., Laaser J.E., Lodge T.P., Reineke T.M. (2016). Tuning Cationic Block Copolymer Micelle Size by pH and Ionic Strength. Biomacromolecules.

[51] Lee H., Son S.H., Sharma R., Won Y.Y. (2011). A Discussion of the pH-Dependent Protonation Behaviors of Poly(2-(Dimethylamino)ethyl Methacrylate) (PDMAEMA) and Poly(Ethylenimine-*ran*-2-ethyl-2-oxazoline) (P(EI-*r*-EOz)). J. Phys. Chem. B.

[52] Fong C., Zhai J., Drummond C.J., Tran N. (2020). Micellar Fd3m Cubosomes from Monoolein–Long Chain Unsaturated Fatty Acid Mixtures: Stability on Temperature and pH Response. J. Colloid Interface Sci..

[53] Muller F., Salonen A., Glatter O. (2010). Phase Behavior of Phytantriol/Water Bicontinuous Cubic Pn3m Cubosomes Stabilized by Laponite Disc-Like Particles. J. Colloid Interface Sci..

[54] de Campo L., Yaghmur A., Sagalowicz L., Leser M.E., Watzke H., Glatter O. (2004). Reversible Phase Transitions in Emulsified Nanostructured Lipid Systems. Langmuir.

[55] Sagalowicz L., Michel M., Adrian M., Frossard P., Rouvet M., Watzke H.J., Yaghmur A., de Campo L., Glatter O., Leser M.E. (2006). Crystallography of Dispersed Liquid Crystalline Phases Studied by Cryo-Transmission Electron Microscopy. J. Microsc..

[56] Barauskas J., Johnsson M., Joabsson F., Tiberg F. (2005). Cubic Phase Nanoparticles (Cubosome†): Principles for Controlling Size, Structure, and Stability. Langmuir.

[57] Gustafsson J., Ljusberg-Wahren H., Almgren M., Larsson K. (1997). Submicron Particles of Reversed Lipid Phases in Water Stabilized by a Nonionic Amphiphilic Polymer. Langmuir.

[58] Almgren M., Edwards K., Gustafsson J. (1996). Cryo Transmission Electron Microscopy of Thin Vitrified Samples. Curr. Opin. Colloid Interface Sci..

[59] Yaghmur A., de Campo L., Sagalowicz L., Leser M.E., Glatter O. (2005). Emulsified Microemulsions and Oil-Containing Liquid Crystalline Phases. Langmuir.

[60] Johnsson M., Lam Y., Barauskas J., Tiberg F. (2005). Aqueous Phase Behavior and Dispersed Nanoparticles of Diglycerol Monooleate/Glycerol Dioleate Mixtures. Langmuir.

[61] Kuntsche J., Horst J.C., Bunjes H. (2011). Cryogenic Transmission Electron Microscopy (Cryo-TEM) for Studying the Morphology of Colloidal Drug Delivery Systems. Int. J. Pharm..

[62] Pippa N., Merkouraki M., Pispas S., Demetzos C. (2013). DPPC:MPOx Chimeric Advanced Drug Delivery Nano Systems (chi-aDDnSs): Physicochemical and Structural Characterization, Stability and Drug Release Studies. Int. J. Pharm..

[63] Yaghmur A., Laggner P., Almgren M., Rappolt M. (2008). Self-Assembly in Monoelaidin Aqueous Dispersions: Direct Vesicles to Cubosomes Transition. PLoS ONE.

[64] Dong Y.D., Larson I., Barnes T.J., Prestidge C.A., Allen S., Chen X., Roberts C.J., Boyd B.J. (2012). Understanding the Interfacial Properties of Nanostructured Liquid Crystalline Materials for Surface-Specific Delivery Applications. Langmuir.

[65] Balestri A., Lonetti B., Harrisson S., Farias-Mancilla B., Zhang J., Amenitsch H., Schubert U.S., Guerrero-Sanchez C., Montis C., Berti D. (2022). Thermo-Responsive Lipophilic NIPAM-Based Block Copolymers as Stabilizers for Lipid-Based Cubic Nanoparticles. Colloids Surf. B Biointerfaces.

[66] Kulkarni C.V., Vishwapathi V.K., Quarshie A., Moinuddin Z., Page J., Kendrekar P., Mashele S.S. (2017). Self-Assembled Lipid Cubic Phase and Cubosomes for the Delivery of a Model Drug (Aspirin). Langmuir.

