# Supplementary material for: Stimuli-Responsive Cationic Lyotropic Liquid Crystalline Nanoparticles: Formulation Process, Physicochemical and Morphological Evaluation
Source: Pharmaceutics. 2025 Sep 15;17(9):1199. doi: 10.3390/pharmaceutics17091199 (PMC12473691; doi:10.3390/pharmaceutics17091199)
Supplement: Supplementary file 1 [file pharmaceutics-17-01199-s001.zip › pharmaceutics-3756737-supplementary.docx]

**Supporting Information**

**Stimuli-responsive cationic lyotropic liquid crystalline nanoparticles: Formulation process, physicochemical and morphological evaluation**

Maria Chountoulesi^a,*^, Natassa Pippa^a^, Varvara Chrysostomou^a,b^, Aleksander Forys^c^, Barbara Trzebicka^c^, Stergios Pispas^b^, Costas Demetzos^a^

^a^ Section of Pharmaceutical Technology, Department of Pharmacy, School of Health Sciences, National and Kapodistrian University of Athens, Panepistimioupolis Zografou 15771, Athens, Greece

^b^ Theoretical and Physical Chemistry Institute, National Hellenic Research Foundation, 48 Vassileos Constantinou Avenue, 11635 Athens, Greece

^c^ Centre of Polymer and Carbon Materials, Polish Academy of Sciences, 34 ul. M. Curie-Skłodowskiej, Zabrze, Poland

(*) Address for correspondence: mchountoules@pharm.uoa.gr


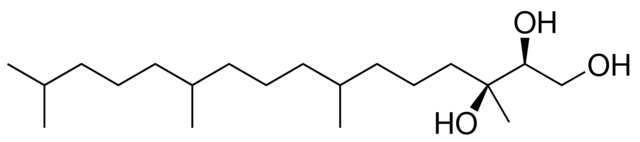


**b.**

**a.**


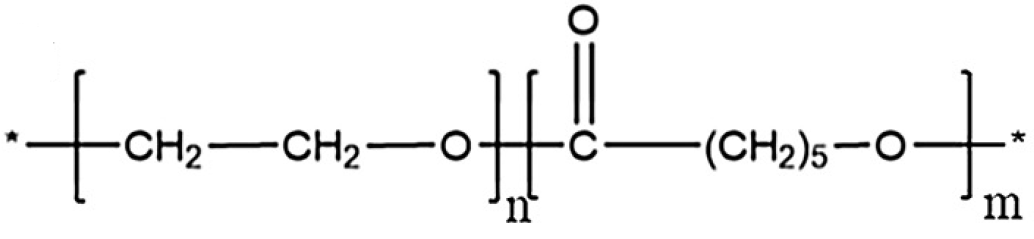


**d.**

**c.**


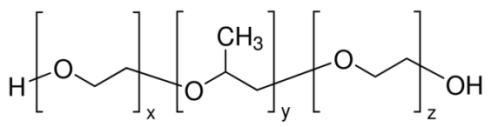


**e.**


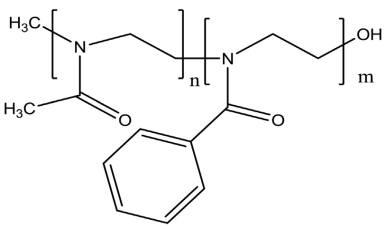


**Figure S1:** Chemical structures of **a.** PHYT lipid, **b.** TPP-QPDMAEMA-b-PLMA block copolymer, **c.** P407 (PEO_98_-PPO_67_-PEO_98_) copolymer, **d.** PEO-b-PCL block copolymers and **e.** MPOx gradient copolymers employed in this study.

**a.**

**b.**

**Figure S2:** Stability assessment of the hydrodynamic radius (*R_h_*, nm) of the prepared nanosystems over time.

**a.**

**b.**

**Figure S3:** Stability assessment of the polydispersity index (PDI) of the prepared nanosystems over time.

**b.**

**a.**

**b.**

**Figure S4:** Polydispersity index (PDI) of the prepared nanosystems in aqueous media with different pH values.

**a.**

**b.**

**Figure S5:** Polydispersity index (PDI) of the prepared nanosystems vs. temperature.

**Synthesis of TPP-QPDMAEMA-b-PLMA**

The quaternization reaction was accomplished by the following process: in a 50 mL round bottom flask, 0.5 g (0.08 mmol) of PDMAEMA-b-PLMA copolymer was dissolved in 25 mL of dry dichloromethane (CH_2_Cl_2_) (2% w/v) (Sigma-Aldrich) under inert atmosphere (N_2_ flow). Afterward, 0.19 g (0.41 mmol) of 4-bromobutyl triphenyl phosphonium bromide was added to the solution. The reaction was carried out at 35 ^o^C under stirring and inert atmosphere for 48 h. The solvent was removed using a rotary evaporator and the residue was re-dissolved in deionized H_2_O. Subsequently, the reaction product was purified by dialysis against deionized H_2_O for 72 h, utilizing dialysis tubing membranes from regenerated cellulose of MWCO 3,500 (MEMBRA-CEL by SERVA). The final product was isolated using a rotary evaporator and dried under vacuum oven for 48 h at room temperature. The quaternization degree was assessed by proton nuclear magnetic resonance (^1^H NMR) spectroscopy and was found to be 20%.

**Dynamic, Static and Electrophoretic Light Scattering Techniques**

The physicochemical behavior of the prepared nanosystems was characterized by measuring the size (hydrodynamic radius *R_h_*, nm) and the size distribution (polydispersity index, PDI) by utilizing dynamic light scattering (DLS), while the ζ-potential (ζ-pot, mV) of the nanoparticles was measured by electrophoretic light scattering (ELS). In all the measurements, 100 μL of aliquots was diluted 30-fold in HPLC grade water, and the others referred to below by using the same media.

In three different media (HPLC-grade water with pH=6.0, phosphate buffered saline, PBS with pH=7.4 and citrate buffer with pH=4.2), the effect of pH of serum proteins (in fetal bovine serum (FBS) medium), the effect of ionic strength (in NaCl 0.10 N, 0.34 N, 0.51N and PBS 0.154N) and the effect of temperature (25 °C, 37 °C and 55 °C) on the physicochemical behavior of the nanosystems was monitored by using DLS, SLS (static light scattering) and ELS measurements.

Statistical analysis was performed by using Student’s *t*-test and multiple comparisons by using one-way ANOVA. *P*-values < 0.05 were considered statistically significant. The *R_h_*, PDI and ζ-pot values of the nanoparticles were averaged from triplicate measurements and the results were reported as a mean ± standard deviation.

**Entrapment Efficiency (EE)% Determination**

The free resveratrol was separated from the resveratrol entrapped in the liquid crystalline nanoparticles using the ultrafiltration centrifugal method (*Chountoulesi et al., 2020, Eur J. Pharm. Biopharm*). In detail, the nanoparticle dispersion was centrifuged for 45 min at 8000 rpm using centrifugal filter tubes [molecular weight (MW) cutoff = 10 kDa; Millipore] at 4°C. The particles were separated from the aqueous phase, and the free resveratrol in the supernatant was analyzed. The UV−Vis spectrophotometry method was used to measure the free drug concentration in the samples after centrifugation and supernatant collection. The absorption measurements were performed at a wavelength of 307 nm using UV−Vis spectrophotometry (Shimadzu PharmaSpec UV-1700 UV/Vis spectrophotometer) and a preconstructed calibration curve. Centrifuged samples of the respective empty liquid crystalline nanoparticles were used as a blank. The entrapment efficiency (EE)% was calculated according to the following equation:

$$\left( EE \right)\%=\left( 1-\frac{C_{supernatant}}{C_{total}} \right)\% (3)$$

where *C_supernatant_* is the resveratrol concentration that was quantified in the supernatant (non-entrapped) and *C_total_* is the total concentration of the resveratrol added in the dispersion (2 mg/mL).

**Table S1**: Encapsulation efficiency (EE%) of the resveratrol.

| **Sample** | **Weight ratio** | **EE %** |
| --- | --- | --- |
| **PHYT:TPP-QPDMAEMA-b-PLMA** | **9:1** | 99.85% |
| **PHYT:TPP-QPDMAEMA-b-PLMA** | **4:1** | 99.72% |
| **PHYT:TPP-QPDMAEMA-b-PLMA:P407** | **8:1:1** | 99.20% |
| **PHYT:TPP-QPDMAEMA-b-PLMA:PEO-b-PCL H1** | **8:1:1** | 99.74% |
| **PHYT:TPP-QPDMAEMA-b-PLMA:PEO-b-PCL H4** | **8:1:1** | 99.65% |
| **PHYT:TPP-QPDMAEMA-b-PLMA:MPOx1** | **8:1:1** | 99.49% |
| **PHYT:TPP-QPDMAEMA-b-PLMA:MPOx2** | **8:1:1** | 99.53% |
